# Coverage Bias and Sensitivity of Variant Calling for Four Whole-genome Sequencing Technologies

**DOI:** 10.1371/journal.pone.0066621

**Published:** 2013-06-11

**Authors:** Nora Rieber, Marc Zapatka, Bärbel Lasitschka, David Jones, Paul Northcott, Barbara Hutter, Natalie Jäger, Marcel Kool, Michael Taylor, Peter Lichter, Stefan Pfister, Stephan Wolf, Benedikt Brors, Roland Eils

**Affiliations:** 1 Division of Theoretical Bioinformatics, German Cancer Research Center (DKFZ), Heidelberg, Germany; 2 Division of Molecular Genetics, German Cancer Research Center (DKFZ), Heidelberg, Germany; 3 Genomics and Proteomics Core Facility, High Throughput Sequencing Unit, German Cancer Research Center (DKFZ), Heidelberg, Germany; 4 Division of Pediatric Neurooncology, German Cancer Research Center (DKFZ), Heidelberg, Germany; 5 The Arthur and Sonia Labatt Brain Tumor Research Centre, The Hospital for Sick Children Research Institute, University of Toronto, Ontario, Canada; 6 Division of Neurosurgery, The Hospital for Sick Children, University of Toronto, Ontario, Canada; 7 Department of Pediatric Hematology and Oncology, Heidelberg University Hospital, Heidelberg, Germany; 8 Department of Bioinformatics and Functional Genomics, Institute of Pharmacy and Molecular Biotechnology, and Bioquant, University of Heidelberg, Heidelberg, Germany; Harvard School of Public Health, United States of America

## Abstract

The emergence of high-throughput, next-generation sequencing technologies has dramatically altered the way we assess genomes in population genetics and in cancer genomics. Currently, there are four commonly used whole-genome sequencing platforms on the market: Illumina’s HiSeq2000, Life Technologies’ SOLiD 4 and its completely redesigned 5500xl SOLiD, and Complete Genomics’ technology. A number of earlier studies have compared a subset of those sequencing platforms or compared those platforms with Sanger sequencing, which is prohibitively expensive for whole genome studies. Here we present a detailed comparison of the performance of all currently available whole genome sequencing platforms, especially regarding their ability to call SNVs and to evenly cover the genome and specific genomic regions. Unlike earlier studies, we base our comparison on four different samples, allowing us to assess the between-sample variation of the platforms. We find a pronounced GC bias in GC-rich regions for Life Technologies’ platforms, with Complete Genomics performing best here, while we see the least bias in GC-poor regions for HiSeq2000 and 5500xl. HiSeq2000 gives the most uniform coverage and displays the least sample-to-sample variation. In contrast, Complete Genomics exhibits by far the smallest fraction of bases not covered, while the SOLiD platforms reveal remarkable shortcomings, especially in covering CpG islands. When comparing the performance of the four platforms for calling SNPs, HiSeq2000 and Complete Genomics achieve the highest sensitivity, while the SOLiD platforms show the lowest false positive rate. Finally, we find that integrating sequencing data from different platforms offers the potential to combine the strengths of different technologies. In summary, our results detail the strengths and weaknesses of all four whole-genome sequencing platforms. It indicates application areas that call for a specific sequencing platform and disallow other platforms. This helps to identify the proper sequencing platform for whole genome studies with different application scopes.

## Introduction

Massively parallel sequencing (next-generation sequencing) has revolutionized research in cancer genetics and genomics [1] and enhanced our understanding of natural human genetic variation [2], [3]. Lam *et al.* [4] have performed a detailed comparison of two next-generation sequencing technologies, Illumina’s HiSeq2000 and Complete Genomics, with respect to their sensitivity to call single nucleotide variants (SNV) and indels. Other studies provided insight into technology-specific error profiles [5], [6] and concordance between different platforms [7]. These studies, however, were based on comparing Life Technologies’ SOLiD and Illumina’s GAII with pyrosequencing or Sanger sequencing [8], [9], [10], the costs of which are prohibitive for whole-genome sequencing studies in mammals. These comparative studies have been performed either on a global scale [4], [7] or for CpG islands that are less well assessed by next-generation sequencing methods [11], a well-known phenomenon called GC bias [12], [13], [14].

Here, we sequenced two tumor/normal pairs obtained from two pediatric medulloblastoma patients (MB14/BL14 and MB24/BL24) with at least 30x coverage on all commonly used, state-of-the-art next-generation sequencing platforms for whole genome sequencing, namely Life Technologies’ SOLiD 4 and its completely redesigned 5500xl SOLiD, Illumina’s HiSeq2000, and Complete Genomics’ technology (Table 1). We then compared their ability to call SNVs in whole-genome sequencing data with high confidence. As gold standard for SNV calling, we used genotypes determined by Affymetrix SNP 6.0 Array Technology (total of 907,551 SNPs after quality filtering). In addition, we performed a detailed analysis of how evenly each of these technologies covers the entire genome, and how the reads are distributed across 25 specific genomic regions. Finally, we studied how a combination of data from different technologies might help to overcome the limitation or bias in SNV calling by any of the four technologies alone.

**Table 1:** Average coverage information for each sample and platform assessed.

	Complete Genomics	HiSeq2000	SOLiD 4	5500xl SOLiD
**MB14**	45.46x	29.87x	30.0x	-
**BL14**	51.64x	34.06x	30.0x	-
**MB24**	51.76x	34.48x	30.0x	32.51x
**BL24**	50.0x	33.29x	30.0x	31.0x

## Materials and Methods

### Whole-genome sequencing

We sequenced two tumor/normal pairs obtained from the primary untreated tumor and whole blood of two pediatric medulloblastoma patients (MB14/BL14, female and MB24/BL24, male).

High molecular weight genomic DNA was fragmented in a Covaris instrument (Woburn, MA, USA) to an average size of 400 nucleotides for HiSeq2000 sequencing and of 230 nucleotides for SOLiD sequencing, respectively (Table S1).

HiSeq2000 Library preparation was performed using standard Illumina protocols and Illumina paired-end adapters. For HiSeq2000 sequencing, a PhiX kit v2 library (Illumina) was spiked into the libraries at a proportion of about 1% each. The total loading concentration was 7pM. Amplification was performed in the cBOT (Illumina) using an Illumina TruSeq paired-end v2-cluster generation chemistry. For sequencing, 200 cycle TruSeq-v2-SBS chemistry was used and 2×101 cycles of sequencing were performed. Base calling was performed with Illumina RTA v1.10.36 software.

For Life Technologies’ SOLiD 4 and 5500xl SOLiD sequencing, genomic libraries were prepared following the manufacturer's standard instructions. Emulsion PCRs were performed using SOLiD™ EZ Bead™ Systems.

SOLiD 4 sequencing was performed using Life Technologies standard protocols with 50/35 PE chemistry and model caller version MCC 4.04. 5500xl SOLiD sequencing was carried out using 75/35 PE chemistry following the manufacturers standard protocols and MCC 5500 1.0 software.

### Read mapping and SNV calling

Sequences were aligned to the human reference genome (NCBI build 37/HG19). Due to the heterogeneous nature of the sequencing data, for each platform we used different alignment algorithms. Alignment filters were kept as similar as possible. For HiSeq2000 sequences, we mapped the reads using the Burrows Wheeler Aligner [15] v0.5.9-r16. For SOLiD 4 and 5500xl SOLiD, reads were aligned using Life Technologies’ proprietary Lifescope 2.1 software. Duplicate reads were removed using the Picard software tools v1.61 (http://picard.sourceforge.net/). Base recalibration was performed using the Genome Analysis ToolKit (GATK) [16] v1.3. SNVs were called using samtools [17] v0.1.18 and for Life Technologies’ data in addition we used Lifescope 2.1. However, samtools yielded better results, translating into a larger area under curve (AUC) in the receiver operating characteristic (ROC) curves comparing with the Affymetrix SNP6 array. Complete Genomics performed sequencing and data analysis using their proprietary pipeline (Software v2.0.1.5). Unless otherwise mentioned, all results correspond to 30x mean coverage, or for Complete Genomics to full coverage generated (for details see Table 1).

For validation of SNP calls with an independent technology, Affymetrix SNP 6 arrays were hybridized and analyzed as previously described [18]. Receiver operating characteristic (ROC) curves were computed using coverage at the SNP position as the independent variable. For these we used samtools mpileup with the following settings, generating vcf files split by chromosome ($chrom): -AE was used for HiSeq2000 data, -AB for SOLiD and Complete Genomics data. Several quality cutoffs were tested ($Q: 1 and 13) and the cutoff selected that provided the largest AUC for the comparison with the SNP6 array. For HiSeq2000, additional arguments were (samtools mpileup –R –I –A –E –q 1 –Q $Q –r $chrom –ugf $REF $BAM | bcftools view –vcgNI - | vcfutils.pl varFilter > result.vcf), and for the SOLiD platforms and Complete Genomics the following command was used: (samtools mpileup –R –I –A –B –q 1 –Q $Q –r $chrom –ugf $REF $BAM | bcftools view –vcgNI - | vcfutils.pl varFilter > result.vcf).

### Coverage and downsampling

Average base coverage was computed after duplicate removal for all informative bases of the reference genome (excluding Ns) using a custom script. For downsampling we randomly removed read pairs or singletons to reach 30x or 15x mean coverage.

Because the Complete Genomics Analysis Pipeline is not publicly available, we could not downsample the entire data for direct SNV comparison. Complete Genomics mapping files include reads mapped (‘initial mapping files’) and reads mapped by assembly at candidate regions deviating from the reference (‘evidence files’). Thus, for downsampling to 30x we only used the initial mapping files.

### Conversion of Complete Genomics data

Initial mapping files and evidence files were converted to the BAM format using the Complete Genomics Analysis Tools (http://www.completegenomics.com/analysis-tools/cgatools/) v1.5.0.31, then merged and sorted with samtools. Duplicates were removed using the Picard tool v1.61.

### Combination of sequencing data from different technologies

For the combination of data from different technologies, we merged their aligned reads into one BAM file (after base quality recalibration with GATK) and called the variants using samtools mpileup applying -AB, disabling Illumina-specific probabilistic realignment (samtools mpileup –R –I –A –B –q 1 –Q $Q –r $chrom –ugf $REF $BAM | bcftools view –vcgNI - | vcfutils.pl varFilter > result.vcf).

### Coverage distribution and regions without coverage

We computed the per-base coverage and the regions without coverage from BAM files using samtools mpileup. Only uniquely mapping reads were considered. Reference genome regions composed of undefined bases (Ns) as well as chr Y were not considered in our analyses.

Unless otherwise mentioned, a base was considered not covered if it was supported by less than three reads. The rationale behind this cutoff is that we argue 3 reads are the absolute minimum required to call a heterozygous variant - two reads with a non-reference base (to exclude sequence artifacts affecting only one read) and one with the reference base. Base coverage in 1 kb windows was computed as the sum of the coverage per base.

### Functional regions

BED files with the genomic coordinates for CpG islands, CpG island shores, exons, segmental duplications, self chains (downloaded on 09/21/2011), promoters, repeats and mammal conservation (downloaded on 12/19/2011) were downloaded from the UCSC Genome Bioinformatics Site (http://genome.ucsc.edu/).

CpG island shores were defined as 2 kb upstream and downstream of CpG islands [19]. Promoters were defined as 2 kb upstream and 500 bp downstream from the transcription start site. Intron coordinates were generated from Exon coordinates using a custom Perl script and BEDTools [20] v2.14.3. BED files for different subcategories of repeats were generated by splitting the UCSC repeats file according to repeat type.

The coordinates for the Cancer Gene Census (downloaded on 05/31/2011) and genes from the Cosmic database (downloaded on 11/09/2011) are from the Wellcome Trust Sanger Institute (http://www.sanger.ac.uk/). Overlaps with regions without coverage were computed with BEDTools.

### Statistical tests

For the pairwise platform comparisons of GC bias, we used Kolmogorov-Smirnov tests for GC percentages below 25% and above 60%. Coverage input values were sampled from the loess curves.

For the comparison between platforms of the coverage distribution and the comparison between platforms of the fraction without coverage for specific genomic regions, we used two-sample Student’s t-tests. For the comparison of the ROC curves, we focused on the sensitivity, comparing the sensitivity between different technologies and samples with paired two-sample Student's t-tests.

Differences yielding p-values below or equal to 0.05 were considered significant. We did not compute p-values for 5500xl SOLiD because of the small sample size (two samples).

### Data access

All short-read sequencing data have been deposited at the European Genome-phenome Archive (EGA, http://www.ebi.ac.uk/ega/), which is hosted by the EBI, under accession number EGAS00001000274. The Affymetrix SNP6 array data has been deposited at Array Express (http://www.ebi.ac.uk/arrayexpress/) under accession number E-MTAB-1159.

Custom scripts, variant calls and the BED annotation data are available under https://ibios.dkfz.de/documents/rieber/scripts_and_annotation.

### Ethics statement

All patient material for this study was collected after obtaining written informed consent from participants and an ethical vote approving the study (Institutional Review Board: Ethics Committee of the Medical Faculty of Heidelberg University, Germany/Ethikkommission der Medizinischen Fakultät Heidelberg) according to ICGC guidelines (www.icgc.org).

## Results

### GC bias

We first assessed the sequencing quality of each platform with respect to distributing reads most evenly across the genome. GC bias describes the dependence between coverage and GC content, where both GC-rich and GC-poor regions are less well covered than regions with balanced base composition. Ideally, with no GC bias present, we would see a uniform distribution of coverage, independent of GC content.

**Figure 1:**
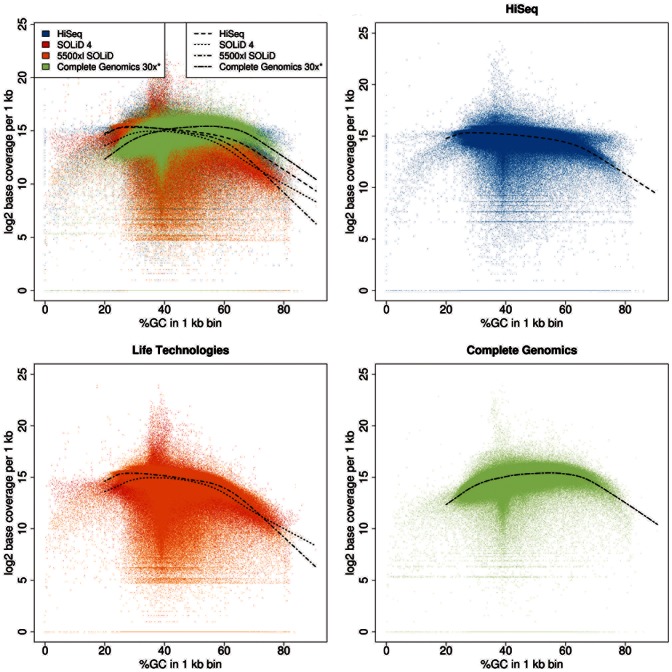
GC bias for each platform. Log2 base coverage in 1 kb windows versus GC content for HiSeq2000, SOLiD 4, 5500xl SOLiD, and Complete Genomics data. The first panel shows an overlay of all four technologies. The upper right panel shows HiSeq2000 only (blue), the lower left SOLiD 4 and 5500xl SOLiD (red and orange, respectively), and the lower right Complete Genomics at downsampled 30x coverage (light green). Smoothed loess curves are fitted to each dataset to represent the local coverage trend. Exemplary data from patient sample MB24 is shown.

When comparing the GC bias of the different technologies (Figure 1, Figure S1–S4), we found significant differences between all platforms, except for SOLiD 4 vs. HiSeq2000 for a GC percentage above 60%. P-values from Kolmogorov-Smirnov tests for pairwise platform comparisons of GC bias for patient sample MB24 are listed in Table 2. The most pronounced GC bias is found for Life Technologies’ SOLiD 4 and 5500xl SOLiD, especially in regions with more than 60% GC content. HiSeq2000 shows a slightly reduced GC bias here (significant in two out of four samples: MB14 and BL14). Note that we used v2 chemistry for HiSeq 2000 sequencing of all four samples. The latest release of v3 chemistry does not reveal a dramatic reduction in GC bias compared to the earlier v2 chemistry (Figure S5). The least GC bias for GC rich regions is revealed by Complete Genomics, even when the higher mean coverage of 50x (hereafter, ‘Complete Genomics’), shown in Figure S1–S4, is computationally reduced to 30x mean coverage (hereafter, ‘Complete Genomics 30x’) for comparison reasons. At regions with GC content lower than 25%, 5500xl SOLiD and HiSeq2000 perform similarly with a generally lower bias than SOLiD 4 and Complete Genomics. Complete Genomics performs worst in GC poor regions at downsampled 30x coverage. The GC bias at GC rich and poor regions, respectively, was consistently found across all four sequenced samples (Figure S1–S4), except for patient sample BL14 where HiSeq2000 and Complete Genomics 30x perform similarly (p-value 0.9307 for %GC ≤25% and 0.4755 for %GC ≥ 60%).

**Table 2:** P-values from Kolmogorov-Smirnov tests for pairwise platform comparisons of GC bias for patient sample MB24.

	% GC in 1 kb bin ≤25%	% GC in 1 kb bin ≥60%
**HiSeq2000 – CG 30x**	***0.00217***	***0.03561***
**HiSeq2000 – SOLiD 4**	***0.00217***	**0.0779**
**CG 30x – SOLiD 4**	***0.00217***	***3.964e-05***

CG 30x stands for Complete Genomics downsampled to 30x mean coverage. P-values below 0.05 are highlighted in bold and italic.

### Distribution of coverage

**Figure 2:**
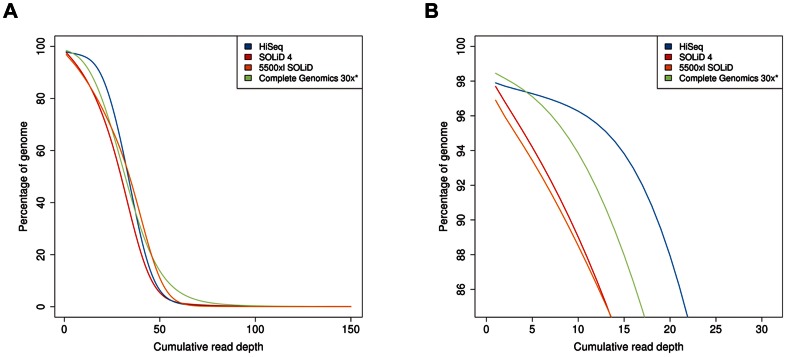
Base coverage distribution for the four platforms. (a) Percentage of genome covered by a given read depth. For each platform, the curve corresponds to the mean of the samples listed in Table 1. (b) Magnified view of the curves shown in a.

We see striking differences between platforms in the distribution of coverage across the genome (Figure 2a and b). At the same mean coverage, SOLiD 4 and 5500xl SOLiD show about 6 times more bases supported by less than 5 reads compared to HiSeq2000 and Complete Genomics (Table 3, averaged across all samples; p-value HiSeq2000 vs. SOLiD 4: 0.001; Complete Genomics 30x vs. SOLiD 4: 0.001). Coverage distribution is similar for SOLiD 4 and 5500xl SOLiD, with 5500xl SOLiD showing a slightly higher number of bases with higher coverage (20–60x). HiSeq2000 shows by far the narrowest coverage distribution compared to all other sequencing platforms. Complete Genomics has the broadest coverage distribution. Even for Complete Genomics downsampled to 30x average coverage, the coverage distribution is still wider than the one resulting from HiSeq2000. The cumulative coverage distribution (Figure 2b) reveals that 5500xl SOLiD covers the smallest percentage of the genome, while HiSeq2000 and SOLiD 4 cover a similar and slightly higher fraction. However, the genomic coverage of all three platforms is exceeded by Complete Genomics at both 30x and 50x (see also Figure S6).

**Table 3:** Number of bases covered on average across all samples from Table 1, and average number of bases covered with less than 5 reads, for each platform assessed.

	Complete Genomics	Complete Genomics 30x	HiSeq2000	SOLiD 4	5500xl SOLiD
**Total number of bases covered**	2,826,524,353	2,817,003,995	2,801,114,390	2,795,379,490	2,772,621,192
**Number of bases covered with less than 5 reads**	15,938,617	38,555,229	17,727,532	100,145,774	99,297,132

We further observe higher variations in coverage distribution between samples by Complete Genomics compared to the other platforms, with the fraction of the genome covered with at least 30x differing up to about 15%, and even up to about 18% for the fraction of the genome covered with at least 50x (Figure S6). This is probably largely due to the differences in average coverage between Complete Genomics samples (Table 1), but the variation can still be observed to a slightly lesser extent for low cumulative read depth at downsampled 30x coverage.

### Coverage of genomic regions

To further evaluate the coverage differences between the different platforms, we investigated the distribution across genomic and functional regions. Here, we considered bases covered by fewer than three reads as “ not covered” or “without coverage” (see Material and Methods).

**Figure 3:**
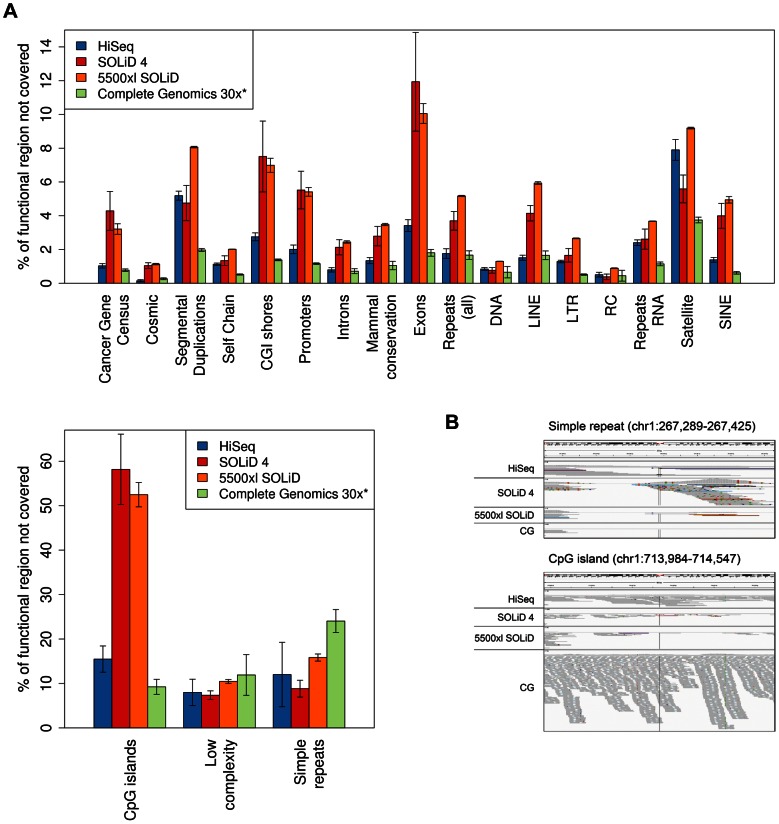
Bases without coverage in different genomic regions. (a) Mean percentage of bases not covered across genomic elements. Bases covered with less than three reads were considered not covered. Note that reducing this threshold to 1 does not dramatically change the overall distribution of reads (Figure S8). Error bars represent one standard deviation as obtained from analyzing all samples listed in Table 1. DNA, LINE, Low complexity, LTR, RC, RNA, Satellite, Simple repeats and SINE are subcategories of Repeats (all). For better visibility, CpG islands, low complexity and simple repeats are plotted separately. (b) Visualization of read coverage for two exemplary genomic regions from patient sample MB24 by IGV for HiSeq2000, SOLiD 4, 5500xl SOLiD and Complete Genomics.

Each of the four technologies has its strengths and weaknesses in covering different sections of the genome (Figure 3a). Complete Genomics shows a uniform coverage of almost all regions with a generally very low percentage (< 2%) of bases not covered, both at 30x coverage and at full coverage (Figure S7). It reveals a comparably smaller covered fraction only for regions containing a large number of short repeats, like simple repeats (24% uncovered at 30x coverage), low complexity repeats (11.9%), CpG islands (9.2%), and satellites (3.7%). Overall, Complete Genomics performs better than all other technologies in this respect, except for simple repeat regions where it is surpassed by all three other platforms. Comparative coverage of an exemplary simple repeat region is shown in Figure 3b. Almost no reads are mapped to this region by Complete Genomics. Read pairs with reads mapping to different chromosomes can be identified in HiSeq2000, SOLiD 4 and 5500xl SOLiD sequences, reflecting the difficulty of mapping reads to repeated sequences also for the latter three technologies. Interestingly, SOLiD 4 shows the highest coverage in this example, but also the largest number of differences from the reference genome.

**Figure 4:**
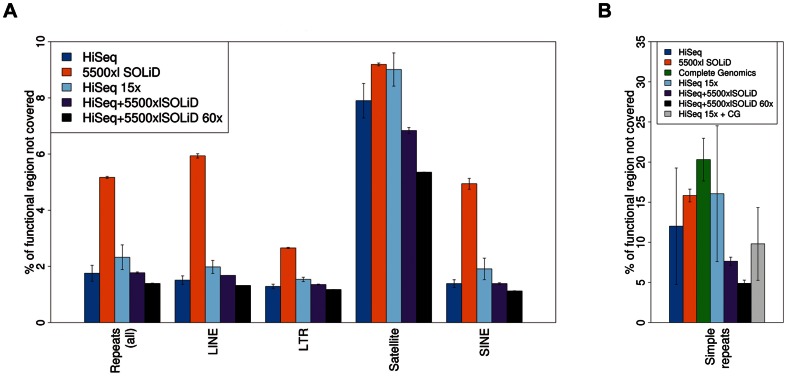
Mean fraction of bases without coverage for different combinations of technologies. (a) Mean fraction of bases not covered for chosen repeat regions. Performance is compared to sequence data from single technology platforms. Only those regions with observable differences are displayed. Error bars represent one standard deviation as obtained from analyzing all samples listed in Table 1. (b) Fraction of bases not covered for simple repeat regions. Error bars represent one standard deviation as obtained from analyzing all samples listed in Table 1.

SOLiD 4 and 5500xl SOLiD sequencing are most affected by GC content and consequently have by far the largest percentage of bases not covered in CpG islands (58.2% and 52.5%, respectively) and CpG island shores (7.5% and 7%, respectively). A t-test yields a p-value of 0.00069 (HiSeq2000 vs. SOLiD 4) and 0.0008 (Complete Genomics 30x vs. SOLiD 4) for CpG islands and a p-value of 0.019 (HiSeq2000 vs. SOLiD 4) and 0.01 (Complete Genomics 30x vs. SOLiD 4) for CpG island shores. We note that for all platforms except for Complete Genomics, the fraction of CpG islands without coverage roughly doubles through our definition of abase not covered (compare to Figure S8), showing that a large proportion of these regions is covered by less than 3 reads. Coverage of an exemplary CpG island is shown in Figure 3b. Complete Genomics shows an impressive coverage of this region followed by HiSeq2000. The lowest coverage is present in SOLiD 4 and 5500xl SOLiD data. Concordant with the differences in coverage of CpG regions, the exome coverage also shows dramatic differences between platforms with a mean difference in the fraction of bases not covered of factor 6.6 between Complete Genomics at 30x and SOLiD 4 (p-value 0.006). Overall, HiSeq2000 performs better than SOLiD 4 and 5500xl SOLiD in nearly all categories except for satellite regions (p-value 0.005 HiSeq2000 vs. SOLiD 4), and even outperforms Complete Genomics (both at full and at 30x coverage) in simple repeat regions (p-value 0.0386). SOLiD 4 performs slightly better than 5500xl SOLiD in repeat regions, while 5500xl SOLiD shows better coverage than SOLiD 4 in most other regions. Interestingly, at the same mean 30x coverage, a combination of HiSeq2000 with 5500xl SOLiD data considerably decreases the fraction not covered of certain repeat regions for both technologies, especially in satellites and simple repeats (Figure 4a and 4b). Similarly, a combination of Complete Genomics data at full coverage with as little as 15x HiSeq2000 data (typically obtained with only one sequencing lane) shows a major increase of covered bases in simple repeats (Figure 4b).

### Regions without coverage

While the number of regions not covered is similar for all platforms for larger sized regions of 150 bp and above (Figure S9), Life Technologies’ platforms SOLiD 4 and 5500xl SOLiD show very high numbers of small regions without coverage compared to HiSeq2000 and Complete Genomics. The smaller the regions, the more pronounced are the differences between platforms, with HiSeq2000 performing better than Complete Genomics. 5500xl SOLiD shows slight improvement over SOLiD 4, except for extremely small regions of 1–2 bp, where this slight difference increases to a drastic difference of a factor of 1.5 in the number of regions not covered (on average 384,304 for SOLiD 4 and 260,252 for 5500xl SOLiD). The fraction of the genome left without coverage (based on the reference genome excluding N’s) at 30x coverage for HiSeq2000 and downsampled Complete Genomics is very similar (1.45% versus 1.61% on average across samples), both performing approximately 2.5 better in this respect than SOLiD 4 and 5500xl SOLiD. At 15x coverage, the difference between HiSeq2000 and the Life Technologies platforms is even more marked with a factor of approximately 3.5, suggesting that the latter can catch up at higher coverage. Notably, Complete Genomics at full coverage leaves only an average of 0.79% of the genome not covered.

### SNP calling

**Figure 5:**
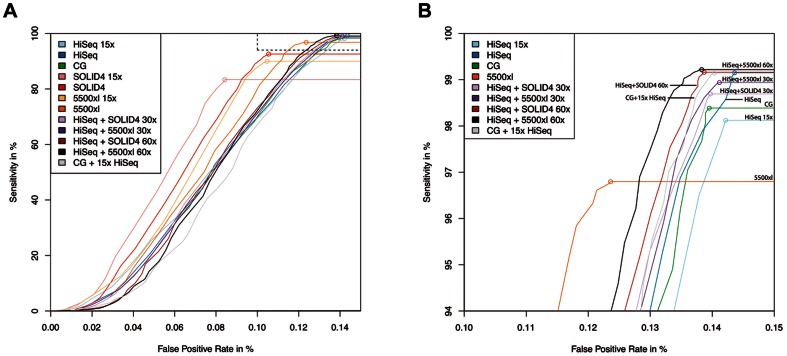
Receiver operating characteristic curves comparing sensitivity and specificity of all sequencing platforms for SNV calling. All curves are computed for exemplary patient sample MB24. When no additional coverage information is indicated, the curves are computed on full coverage data (for coverage information see Table 1). Additional numbers indicate either computationally downsampled data or combined data at specified additive coverage. (a) Specificity plotted from 0–0.15. All curves have reached their plateau at that point and will continue as straight lines. (b) Magnified view of curves as indicated by dashed frame in a) to discriminate between subtle differences in specificity and sensitivity for all curves. Curves that do not appear in this magnified view reached their plateau below the cutoff of 94% sensitivity chosen for this window.

Beyond a mere technological comparison we aimed at estimating the utility of all four sequencing technologies in cancer genome studies, where the major focus is the identification of single nucleotide variants (SNV) with high sensitivity and specificity. There is an ongoing debate on the sensitivity of latest next generation sequencing technologies. As the gold standard for all four samples we used SNPs found by Affymetrix SNP6 arrays as an independent and well-established SNP calling technology (see Figure S10 and Figure S11 for an overview on the distribution of array SNPs in different regions of the genome). It should be noted that the sensitivity for calling SNPs represents an upper bound for calling somatic mutations, since the latter often display mutant allele fractions less than 50%. The SNP calling performance of the different sequencing technologies was compared based on state-of-the art SNV callers using the array data as reference. Using a threshold of increasing coverage to consider a variant ‘called’, we generated receiver operating characteristic (ROC) curves revealing the sensitivity and false positive rate for each technology platform (Figure 5). First, we noticed that a tiny fraction of 0.025% of all SNPs (229/907,551 SNPs) were not correctly identified by any of the four technologies suggesting that those SNPs may not be correctly identified on the arrays.

When comparing all technologies, the overall best sensitivity was achieved by HiSeq2000 (99.15% for sample MB24) followed by Complete Genomics (98.38% sensitivity) (paired t-test on percentage sensitivity comparing Complete Genomics 50x and HiSeq2000 30x, p = 0.008651). Surprisingly, even at downsampled 15x coverage, HiSeq2000 (98.12%) performs close to Complete Genomics at 50x coverage (paired t-test on percentage sensitivity comparing Complete Genomics 50x and HiSeq2000 15x, p = 0.008476). For Complete Genomics, the coverage at genomic positions corresponding to SNPs on the SNP6 array is on average 40x and therefore lower than the overall average coverage of 51.7x. In contrast, HiSeq2000 shows a slightly higher (32.1x) than average coverage (30.0x) in those positions. Still, this difference in coverage does not account for the finding that HiSeq2000 at downsampled 15x coverage shows a sensitivity similar to Complete Genomics at estimated 40x coverage in those positions. With respect to sensitivity, both Life Technologies’ platforms show a performance clearly inferior to HiSeq2000 and Complete Genomics, with 5500xl SOLiD (96.80%) outperforming SOLiD 4 (92.57%) (paired t-test on percentage sensitivity comparing with HiSeq2000, p = 0.008324, and Complete Genomics, p = 0.008189). In contrast, the two Life Technologies’ platforms are superior to Complete Genomics and HiSeq2000 with respect to specificity, exhibiting a lower false positive rate of approximately 0.105–0.124% for 5500xl SOLiD and SOLiD 4.

### Combination of sequencing technologies

Finally, we investigated whether a combination of sequencing data from different sequencing technologies would help to combine the strengths and to compensate for the weaknesses of the four different platforms. As expected, a combination of 30x coverage HiSeq2000 with 30x coverage 5500xl SOLiD data achieves both a slightly increased sensitivity and specificity compared to any of the other technologies alone (Figure 5b). When restricting the total coverage of the combined data sets to 30x, it is very difficult to outperform HiSeq2000 sequencing alone. The best results are obtained by combining HiSeq2000 with 5500xl SOLiD data both at 15x coverage each. The sensitivity of this combined data set almost reaches the sensitivity of HiSeq2000 at full coverage, while the specificity of this combined data set slightly increases over the specificity of 5500xl. Note, however, that this increase in specificity is minimal (0.0025%) compared to the decrease in sensitivity (0.17%). Interestingly, the performance of Complete Genomics sequencing can be enhanced by adding HiSeq2000 data at 15x coverage, which can currently be obtained by only one lane of HiSeq2000 sequencing (paired t-test on percentage sensitivity comparing Complete Genomics and Complete Genomics + HiSeq2000 15x, p = 0.008692). Here, the sensitivity of Complete Genomics increases by 0.73% at a slightly increased specificity.

## Discussion

In this study, we have examined the differences between the four commonly used whole genome next-generation sequencing platforms, Illumina’s HiSeq2000, Life Technologies’ SOLiD 4 and 5500xl SOLiD, and Complete Genomics’ technology. We showed the strengths and weaknesses of each technology with respect to coverage of the genome, distribution of reads across different genomic regions, and SNV calling sensitivity and specificity. We significantly extended earlier comparative studies by including all presently available whole genome sequencing platforms and by using four different samples to shed light on the sample-to-sample variation in sequencing performance.

In our comparison, we did not consider practical parameters such as the required amount of DNA input, sequencing costs, or time required to complete a whole genome sequencing run (Table S1). Those parameters might be much more important for a particular choice of technology when sample material, e.g., from cancer patients, is limited or when sequencing time is critical, e.g., in a cancer diagnostic setting. Here, Complete Genomics with its extremely high demand for sample material and the much slower Life Technologies’ platforms may render themselves inappropriate for certain applications. However, those parameters tend to change rapidly with new technology updates. Further, we explicitly did not include a comparison of different methods for alignment and for SNP calling in this study. Instead, due to the heterogeneity of the sequencing data from different platforms, we used the methods best adapted to each platform, e.g., for mapping, and used comparable stringency parameters whenever possible.

### Coverage comparison

Coverage is an essential aspect of next-generation sequencing, as higher coverage allows for higher confidence during downstream analyses. In tumor samples, where we frequently encounter variants with a very small allele frequency due to contamination with normal tissue, copy number variation, and tumor heterogeneity, high coverage is essential for accurate detection of these variants with high power. Biases in coverage distribution, like the GC bias, are problematic, especially in analyses focusing on fragment abundance (e.g., copy number estimation, RNA-seq), but are also of importance for DNA sequencing, where variants in GC-rich and GC-poor regions might be missed due to low coverage.

Earlier studies [11], [12] have shown a GC bias in particular for Illumina’s GAII sequencing. Suzuki *et al.* [7] claimed that no striking GC bias could be found for SOLiD and 454 sequencing. Ajay *et al.* [21] noticed a much better representation of the coding exome for Illumina’s HiSeq2000 indicating a noticeable reduction in GC bias compared to Illumina GAII. In contrast to Suzuki *et al.* [7], our results clearly show the most pronounced GC bias for Life Technologies’ SOLiD 4 and 5500xl SOLiD, especially in regions with more than 60% GC content. Lam *et al.* [4] reported a lower GC content (41%) and read depth for Illumina-specific single nucleotide variants (SNVs) compared to the SNVs concordantly found (46% GC content) for both Complete Genomics and Illumina. This finding cannot be explained by a difference in GC content only, because at 41–46% GC content both platforms perform equally well with no significant change in GC bias across this range.

Lam *et al.* [4] pointed out that a less uniform coverage indicates that a higher overall sequencing depth is required to achieve a certain level of coverage for most of the genome. We observed that Complete Genomics data, downsampled to 30x, covers a smaller fraction of the genome than HiSeq2000 up to a cumulative read depth of approximately 40x. This confirms the claim that Complete Genomics requires a higher average coverage compared to HiSeq2000 in order to cover a similar fraction of the genome. Also, we observe a considerable degree of sample-to-sample variation in coverage distribution for Complete Genomics data, which is not found for all the other three platforms. This must not be neglected when comparing variant calls between samples since the sensitivity of variant calling depends on coverage.

We found pronounced differences for the coverage of specific genomic regions between platforms. While we see a significant improvement in coverage of CpG islands for HiSeq2000 compared to Illumina’s earlier GAII technology [11], we find that for all platforms except Complete Genomics, a large fraction of CpG islands is covered with less than 3 reads, which explains the earlier observation that SNVs are difficult to call in less well-covered regions such as CpG islands [11]. At first glance, the ability of Complete Genomics to cover CpG islands very well might appear counterintuitive since Complete Genomics, with its shorter read length, should be less able to cover such repetitive genomic elements. As Benjamini and Speed [12] pointed out, the GC content of the entire fragment (and not just the sequenced parts of it) is essential for the degree of GC bias. Complete genomics embeds the DNA fragments to be sequenced into a larger construct containing adapters and self-assembly sequences such that only about half of the resulting DNA stems from the genome of interest [22]. Thus, the technology is less prone to GC bias (Figure 1), which might account for the better coverage of Complete Genomics in GC-rich regions such as CpG islands.

Lam *et al.* [4] suggest that platform-specific differences in SNV calls for Illumina HiSeq2000 and Complete Genomics might be due to mapping difficulties. Interestingly, the striking difference in coverage of simple repeats and low-complexity repeats gives a straightforward explanation for their observation that enrichment of platform-specific SNVs was particularly evident within those genomic repeats. A combination of sequencing data from two different platforms, as suggested by Nothnagel *et al.* [5] for the reduction of false positives in newly identified SNVs, is only of limited use for combining the strengths in coverage of different genomic regions. We observe a gain in the covered fraction only for very restricted genomic regions such as certain types of repeats.

### SNP comparison

Comparisons of SNV calling by different platforms have resulted in different conclusions. While Suzuki *et al.* [7] reported a similar SNP detection performance by Illumina GA and SOLiD, Lam *et al.* [4] compared the reliability of both concordant and discordant calls between Illumina HiSeq2000 and Complete Genomics. Our comparison on SNP calling shows that the best sensitivity is achieved by HiSeq2000, followed by Complete Genomics, supporting a similar conclusion by Lam *et al.* [4]. Overall, we suggest a preference for HiSeq2000 and Complete Genomics in cancer genome studies where sensitivity for detection of low frequency variants matters most, whereas the two Life Technologies platforms might be better suited when calling SNVs with high specificity. Interestingly, for not correctly called homozygous SNPs by HiSeq2000 the number of no-calls on one allele is approximately in the same range as for those not being called on both alleles (Table S2). In contrast, for Complete Genomics we find a significantly higher number of SNPs not being called on either allele (p  =  2.076454e-48 in a binomial test with probability 0.5 for each group).

Further, our results indicate that a combination of sequencing data from different platforms, as suggested by Lam *et al.* [4], is the best approach for comprehensive variation detection. If budget permits, sequencing genomes with both HiSeq2000 and Complete Genomics allows the combination of HiSeq2000’s strength in sensitivity of SNV calling even at low coverage with Complete Genomics’ strength in uniformly covering the entire genome, offering an interesting potential to boost the strengths of both platforms with considerably low efforts.

Our comparative study reveals that certain technologies should not be used for specific applications like epigenome studies relying on good coverage of CpG sequences, whereas the same technology might be the most suited one for diagnostic applications. In contrast to earlier suggestions [5], a combination of different technology platforms is only advised in specific applications where, e.g., coverage of certain functional regions should be combined with high sensitivity in SNV calling across the entire genome. Finally, the dramatic difference in sensitivity of SNV calling for all four platforms strongly indicates that the design of SNV calling algorithms should be well adjusted towards the particular characteristics and level of expected sensitivity of each sequencing platform.

## Supporting Information

Figure S1 **GC bias for each platform for sample MB24, including Complete Genomics at full coverage.** Log2 base coverage in 1 kb windows versus GC content for HiSeq2000, SOLiD 4, 5500xl SOLiD, and Complete Genomics data. The first panel shows an overlay of all four technologies. The upper right panel shows HiSeq2000 only (blue), the lower left SOLiD 4 and 5500xl SOLiD (red and orange, respectively), and the lower right Complete Genomics at full and downsampled 30x coverage (green and light green). Smoothed loess curves are fitted to each dataset to represent the local coverage trend.
(TIFF)

Figure S2 **GC bias for each platform for sample BL24, including Complete Genomics at full coverage.** Log2 base coverage in 1 kb windows versus GC content for HiSeq2000, SOLiD 4, 5500xl SOLiD, and Complete Genomics data. The first panel shows an overlay of all four technologies. The upper right panel shows HiSeq2000 only (blue), the lower left SOLiD 4 and 5500xl SOLiD (red and orange, respectively), and the lower right Complete Genomics at full and downsampled 30x coverage (green and light green). Smoothed loess curves are fitted to each dataset to represent the local coverage trend.
(TIFF)

Figure S3 **GC bias for each platform for sample MB14, including Complete Genomics at full coverage.** Log2 base coverage in 1 kb windows versus GC content for HiSeq2000, SOLiD 4, 5500xl SOLiD, and Complete Genomics data. The first panel shows an overlay of all four technologies. The upper right panel shows HiSeq2000 only (blue), the lower left SOLiD 4 (red), and the lower right Complete Genomics at full and downsampled 30x coverage (green and light green). Smoothed loess curves are fitted to each dataset to represent the local coverage trend.
(TIFF)

Figure S4 **GC bias for each platform for sample BL14, including Complete Genomics at full coverage.** Log2 base coverage in 1 kb windows versus GC content for HiSeq2000, SOLiD 4, 5500xl SOLiD, and Complete Genomics data. The first panel shows an overlay of all four technologies. The upper right panel shows HiSeq2000 only (blue), the lower left SOLiD 4 (red), and the lower right Complete Genomics at full and downsampled 30x coverage (green and light green). Smoothed loess curves are fitted to each dataset to represent the local coverage trend.
(TIFF)

Figure S5 **GC bias for HiSeq2000 with v2 chemistry versus HiSeq2000 with v3 chemistry.** Log2 base coverage in 1 kb windows versus GC content. Smoothed loess curves are fitted to each dataset to represent the local coverage trend. Exemplary data from patient sample MB24 (v2, blue) is compared to another medulloblastoma patient sample (v3, red).
(TIFF)

Figure S6 **Cumulative base coverage distribution for the four platforms for all samples listed in** **Table 1** **.** Percentage of genome covered by read depth. Each curve corresponds to one sample.
(TIFF)

Figure S7 **Percentage of bases without coverage across genomic elements, including Complete Genomics at full coverage.** A base is considered not covered when it is covered by less than three reads. The error bars represent one standard deviation as obtained from analyzing the samples as listed in Table 1. DNA, LINE, Low complexity, LTR, RC, RNA, Satellite, Simple repeats and SINE are subcategories of Repeats (all).
(TIF)

Figure S8 **Percentage of bases without coverage across genomic elements.** In this case, a base is considered not covered when it is covered by zero reads. The error bars represent one standard deviation as obtained from analyzing the samples as listed in Table 1. DNA, LINE, Low complexity, LTR, RC, RNA, Satellite, Simple repeats and SINE are subcategories of Repeats (all).
(TIF)

Figure S9 **Size distribution of regions without coverage for all platforms and samples listed in** **Table 1**. Each curve corresponds to one sample. Based on the reference genome excluding N’s. A base is considered not covered when it is covered by less than three reads. The size of the largest region without coverage is approximately 110,000 bp in size for all four platforms, except for HiSeq (766,173 bp). This is due to the pseudoautosomal region on chrX/Y and is a consequence of mapping differences.
(TIFF)

Figure S10 **Distribution of Affymetrix SNP6 array SNPs in genomic elements analyzed.** Percentage of genome covered by different types of genomic elements, in comparison to the distribution of SNP6 array SNPs on these genomic elements.
(TIFF)

Figure S11 **Distribution of Affymetrix SNP6 array SNPs in repeat types analyzed.** The size of the different repeat regions was analyzed in comparison to the total repeat size. Overlapping repeat regions were reduced and not counted twice. All SNPs mapping to the repeat regions were identified and their distribution across the different repeat types compared to the total number of SNPs.
(TIFF)

Figure S12 **Receiver operating characteristic curves comparing sensitivity and specificity of all sequencing platforms for SNV calling.** All curves are computed for exemplary patient sample BL24. When no additional coverage information is indicated, the curves are computed on full coverage data (for coverage information see Table 1). Additional numbers indicate either computationally downsampled data or combined data at specified additive coverage. (a) Specificity plotted from 0–0.17. All curves have reached their plateau at that point and will continue as straight lines. (b) Magnified view of curves to discriminate between subtle differences in specificity and sensitivity for all curves. Curves that do not appear in this magnified view reached their plateau below the cutoff of 94% sensitivity chosen for this window.
(TIFF)

Figure S13 **Receiver operating characteristic curves comparing sensitivity and specificity of all sequencing platforms for SNV calling.** All curves are computed for exemplary patient sample BL14. When no additional coverage information is indicated, the curves are computed on full coverage data (for coverage information see Table 1). Additional numbers indicate either computationally downsampled data or combined data at specified additive coverage. (a) Specificity plotted from 0–0.17. All curves have reached their plateau at that point and will continue as straight lines. (b) Magnified view of curves to discriminate between subtle differences in specificity and sensitivity for all curves. Curves that do not appear in this magnified view reached their plateau below the cutoff of 94% sensitivity chosen for this window.
(TIFF)

Figure S14 **Receiver operating characteristic curves comparing sensitivity and specificity of all sequencing platforms for SNV calling.** All curves are computed for exemplary patient sample MB14. When no additional coverage information is indicated, the curves are computed on full coverage data (for coverage information see Table 1). Additional numbers indicate either computationally downsampled data or combined data at specified additive coverage. (a) Specificity plotted from 0–0.17. All curves have reached their plateau at that point and will continue as straight lines. (b) Magnified view of curves to discriminate between subtle differences in specificity and sensitivity for all curves. Curves that do not appear in this magnified view reached their plateau below the cutoff of 94% sensitivity chosen for this window.
(TIFF)

Table S1 **Run information for each platform.** Throughput information was obtained from the manufacturer’s homepages.
(XLS)

Table S2 **Detailed comparison of sequencing-based genotype calls with array-based results.** The SNP calls by the SNP6 array were compared based on the genotype level. Splitting into homozygous (hom) and heterozygous (het) array-based calls the sequencing-based results were called as identical (calls on both alleles identical), one_identical (calls only on one allele identical), no_identical (calls on none of the alleles identical) and NA (missing call by array), respectively. Whenever the sequencing data did not show any calls at a given position, we assumed the same genotype at this position as for the reference genome. CG stands for Complete Genomics.
(XLS)

Table S3 **Parameters tested while optimizing samtools mpileup based SNP calling.** The SNP calls for each of the data sets (except for Complete Genomics data) were optimized using the described combinations and the calls providing the strongest overlap with the Affymetrix SNP6 based SNP calls were selected.
(XLS)

Table S4 **Comparison of SNP calling sensitivity.** Tested by paired two-sample t-test.
(XLS)
