# Supplementary figures and images for: Coverage Bias and Sensitivity of Variant Calling for Four Whole-genome Sequencing Technologies
Source: PLoS One. 2013 Jun 11;8(6):e66621. doi: 10.1371/journal.pone.0066621 (PMC3679043; doi:10.1371/journal.pone.0066621)

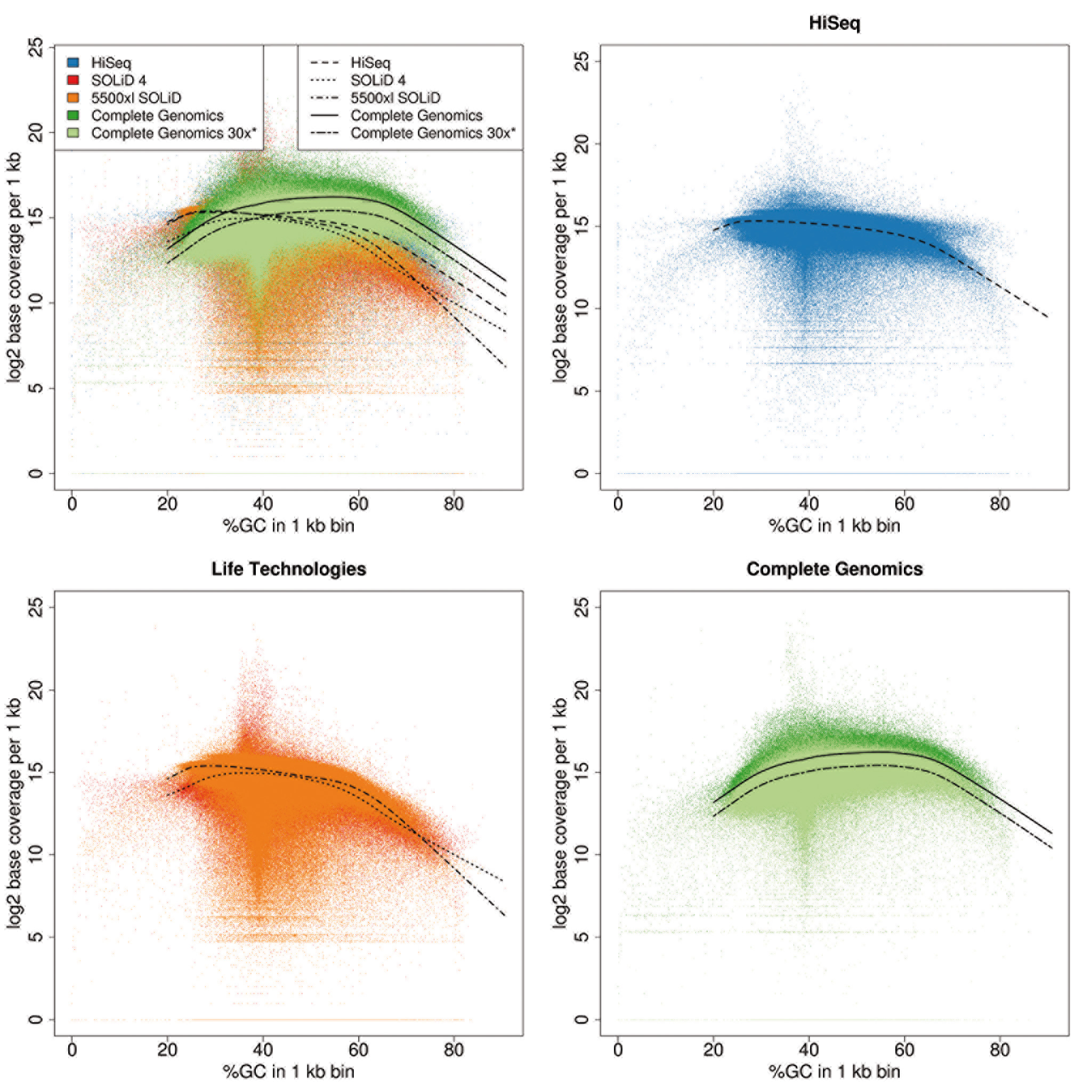

Supplement: Figure S1 — GC bias for each platform for sample MB24, including Complete Genomics at full coverage. Log2 base coverage in 1 kb windows versus GC content for HiSeq2000, SOLiD 4, 5500xl SOLiD, and Complete Genomics data. The first panel shows an overlay of all four technologies. The upper right panel shows HiSeq2000 only (blue), the lower left SOLiD 4 and 5500xl SOLiD (red and orange, respectively), and the lower right Complete Genomics at full and downsampled 30x coverage (green and light green). Smoothed loess curves are fitted to each dataset to represent the local coverage trend. (TIFF) [file pone.0066621.s001.tiff]

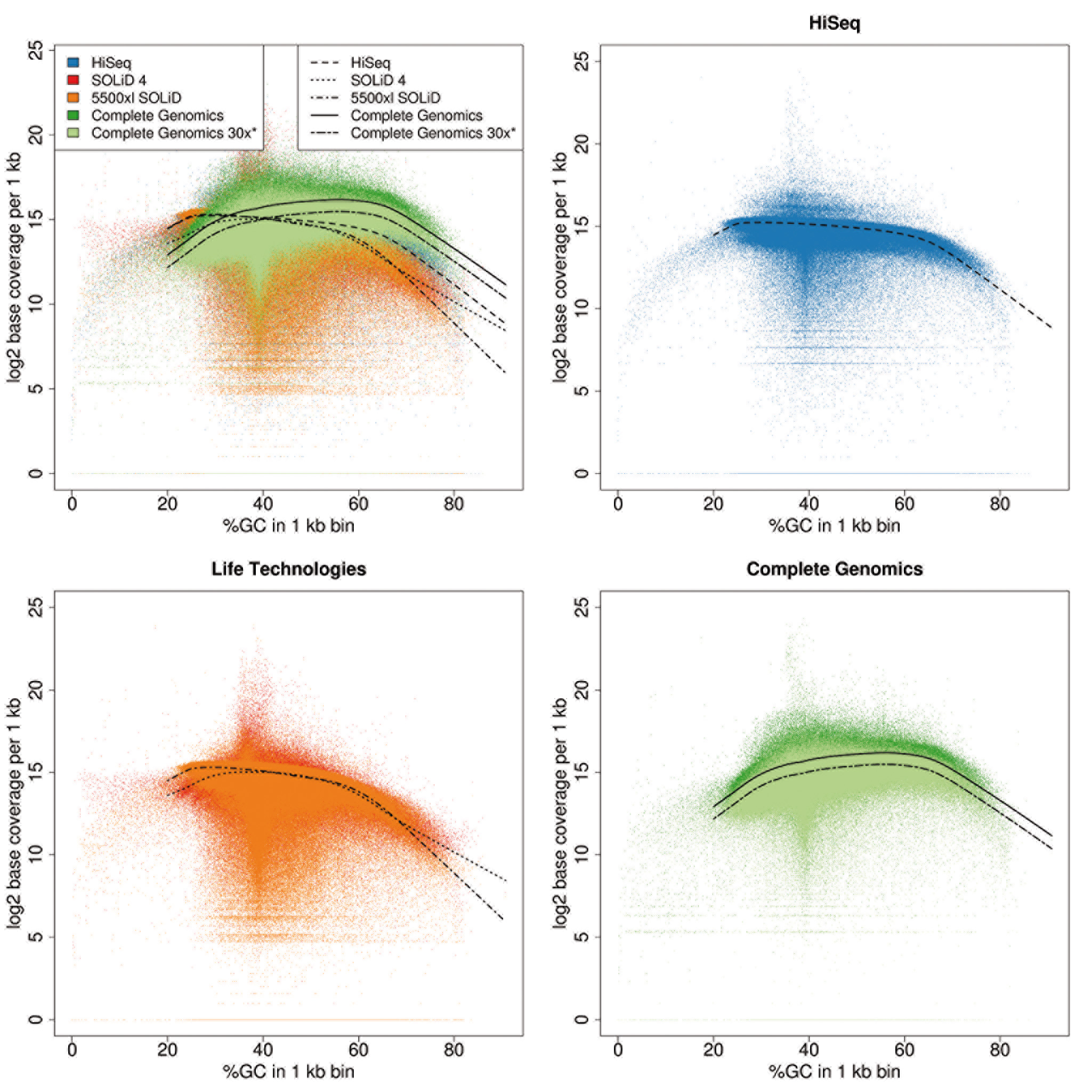

Supplement: Figure S2 — GC bias for each platform for sample BL24, including Complete Genomics at full coverage. Log2 base coverage in 1 kb windows versus GC content for HiSeq2000, SOLiD 4, 5500xl SOLiD, and Complete Genomics data. The first panel shows an overlay of all four technologies. The upper right panel shows HiSeq2000 only (blue), the lower left SOLiD 4 and 5500xl SOLiD (red and orange, respectively), and the lower right Complete Genomics at full and downsampled 30x coverage (green and light green). Smoothed loess curves are fitted to each dataset to represent the local coverage trend. (TIFF) [file pone.0066621.s002.tiff]

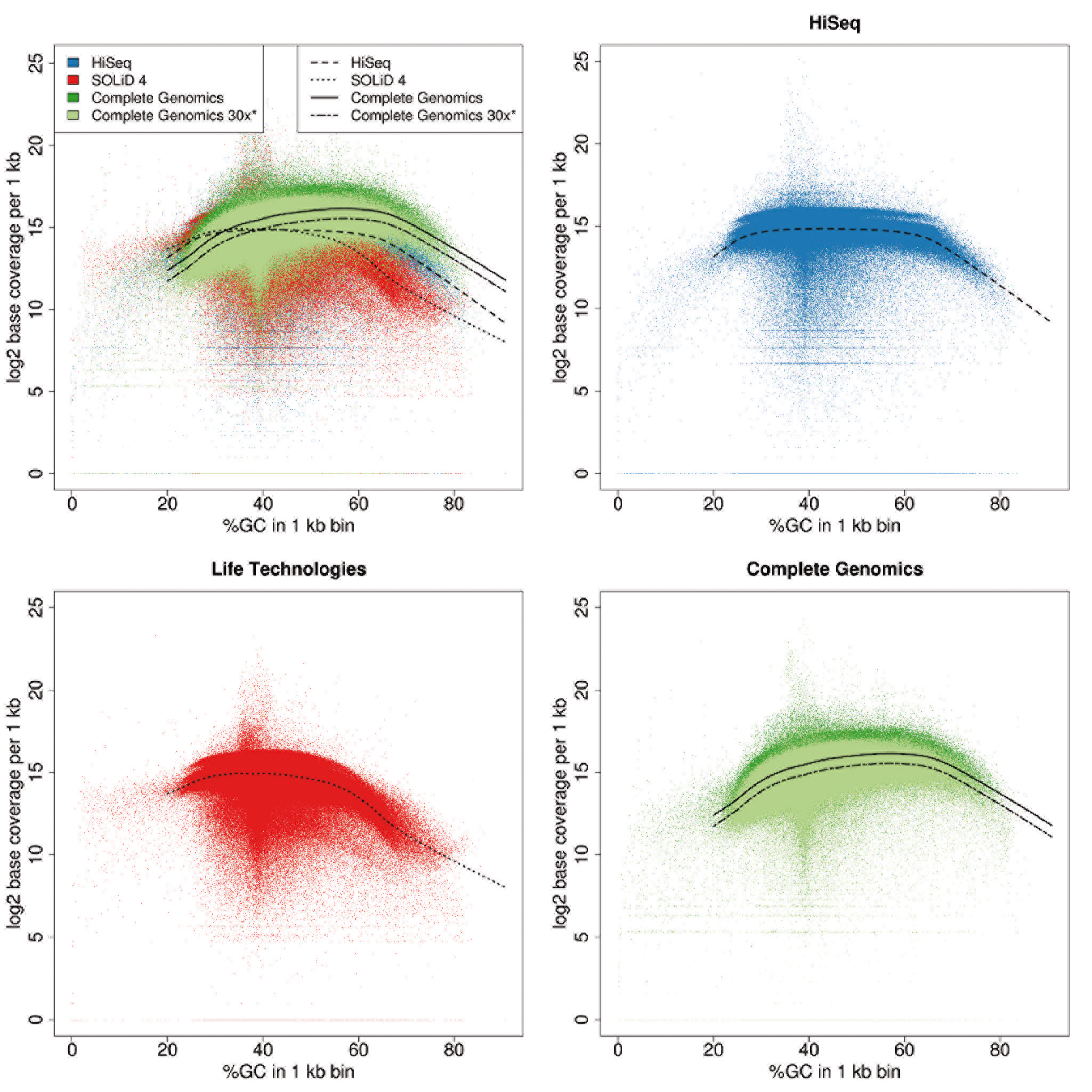

Supplement: Figure S3 — GC bias for each platform for sample MB14, including Complete Genomics at full coverage. Log2 base coverage in 1 kb windows versus GC content for HiSeq2000, SOLiD 4, 5500xl SOLiD, and Complete Genomics data. The first panel shows an overlay of all four technologies. The upper right panel shows HiSeq2000 only (blue), the lower left SOLiD 4 (red), and the lower right Complete Genomics at full and downsampled 30x coverage (green and light green). Smoothed loess curves are fitted to each dataset to represent the local coverage trend. (TIFF) [file pone.0066621.s003.tiff]

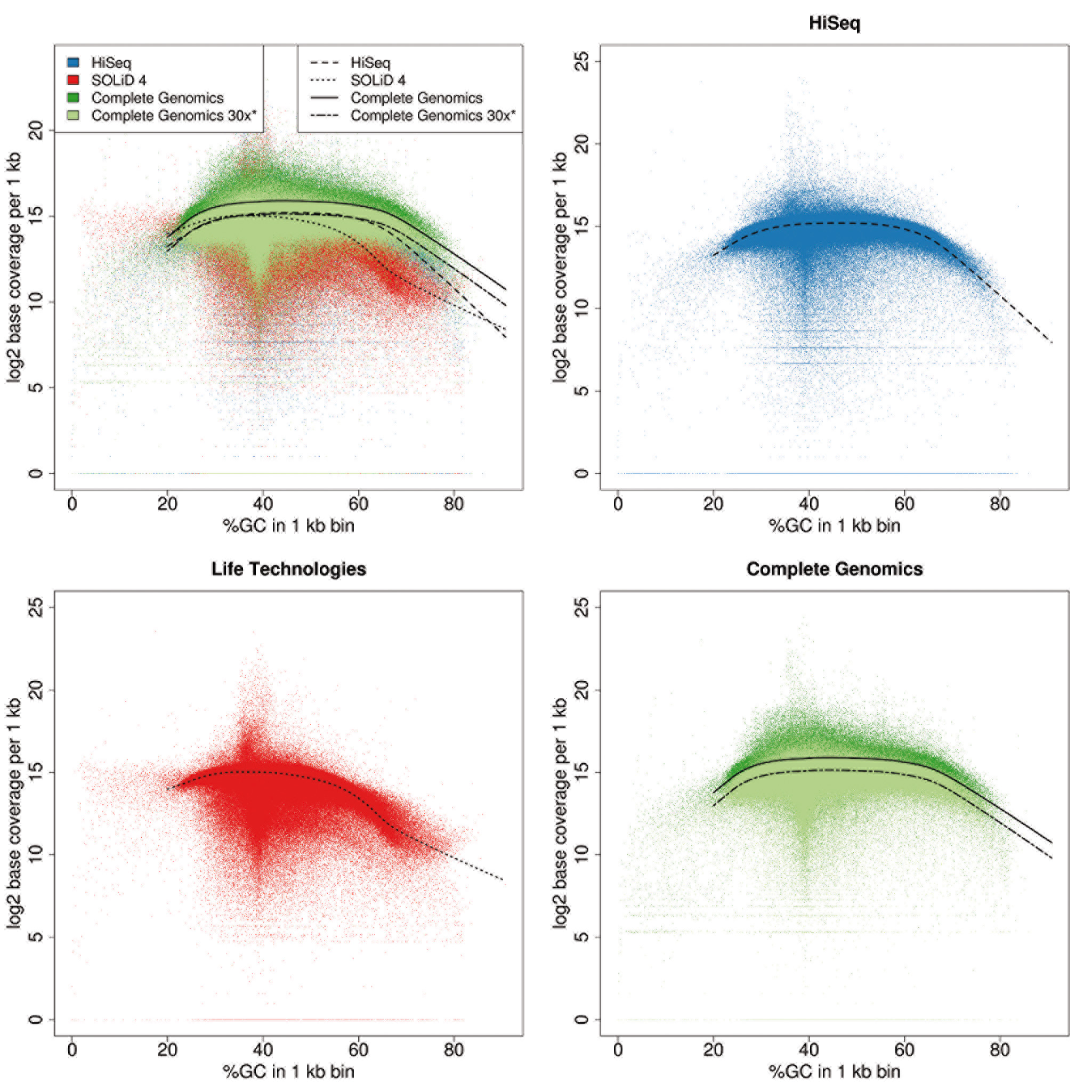

Supplement: Figure S4 — GC bias for each platform for sample BL14, including Complete Genomics at full coverage. Log2 base coverage in 1 kb windows versus GC content for HiSeq2000, SOLiD 4, 5500xl SOLiD, and Complete Genomics data. The first panel shows an overlay of all four technologies. The upper right panel shows HiSeq2000 only (blue), the lower left SOLiD 4 (red), and the lower right Complete Genomics at full and downsampled 30x coverage (green and light green). Smoothed loess curves are fitted to each dataset to represent the local coverage trend. (TIFF) [file pone.0066621.s004.tiff]

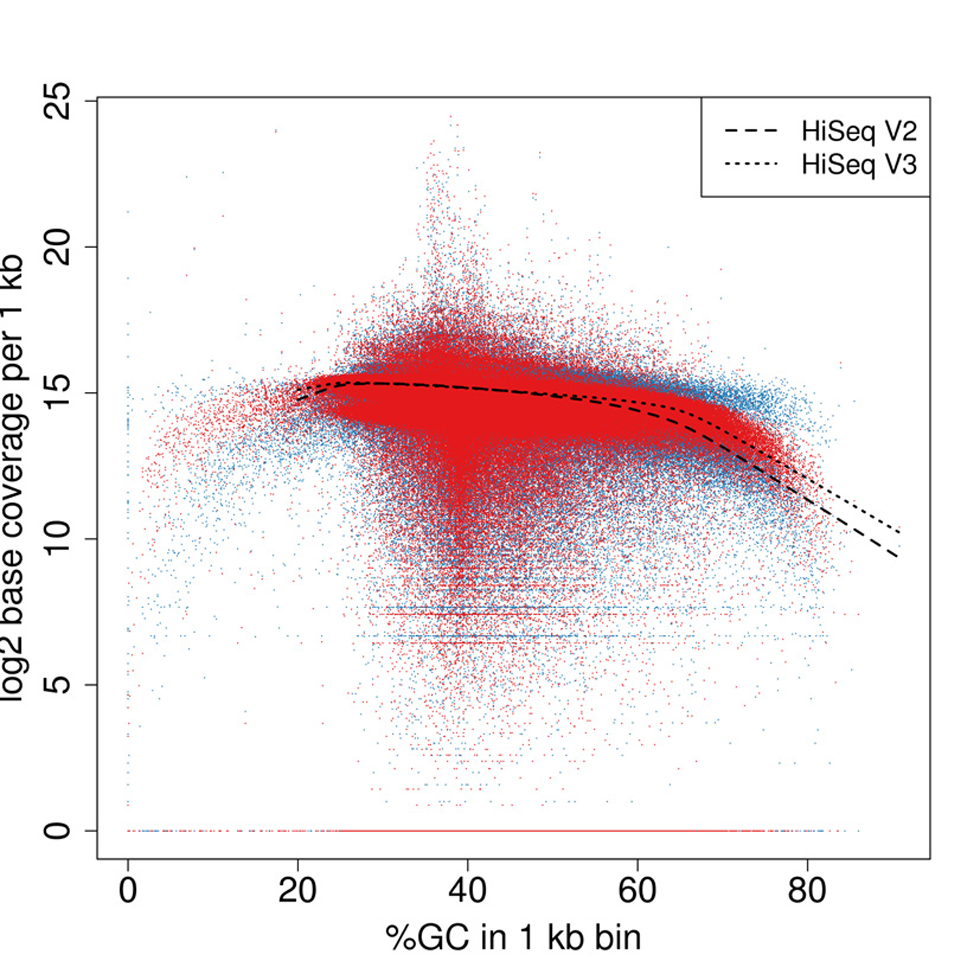

Supplement: Figure S5 — GC bias for HiSeq2000 with v2 chemistry versus HiSeq2000 with v3 chemistry. Log2 base coverage in 1 kb windows versus GC content. Smoothed loess curves are fitted to each dataset to represent the local coverage trend. Exemplary data from patient sample MB24 (v2, blue) is compared to another medulloblastoma patient sample (v3, red). (TIFF) [file pone.0066621.s005.tiff]

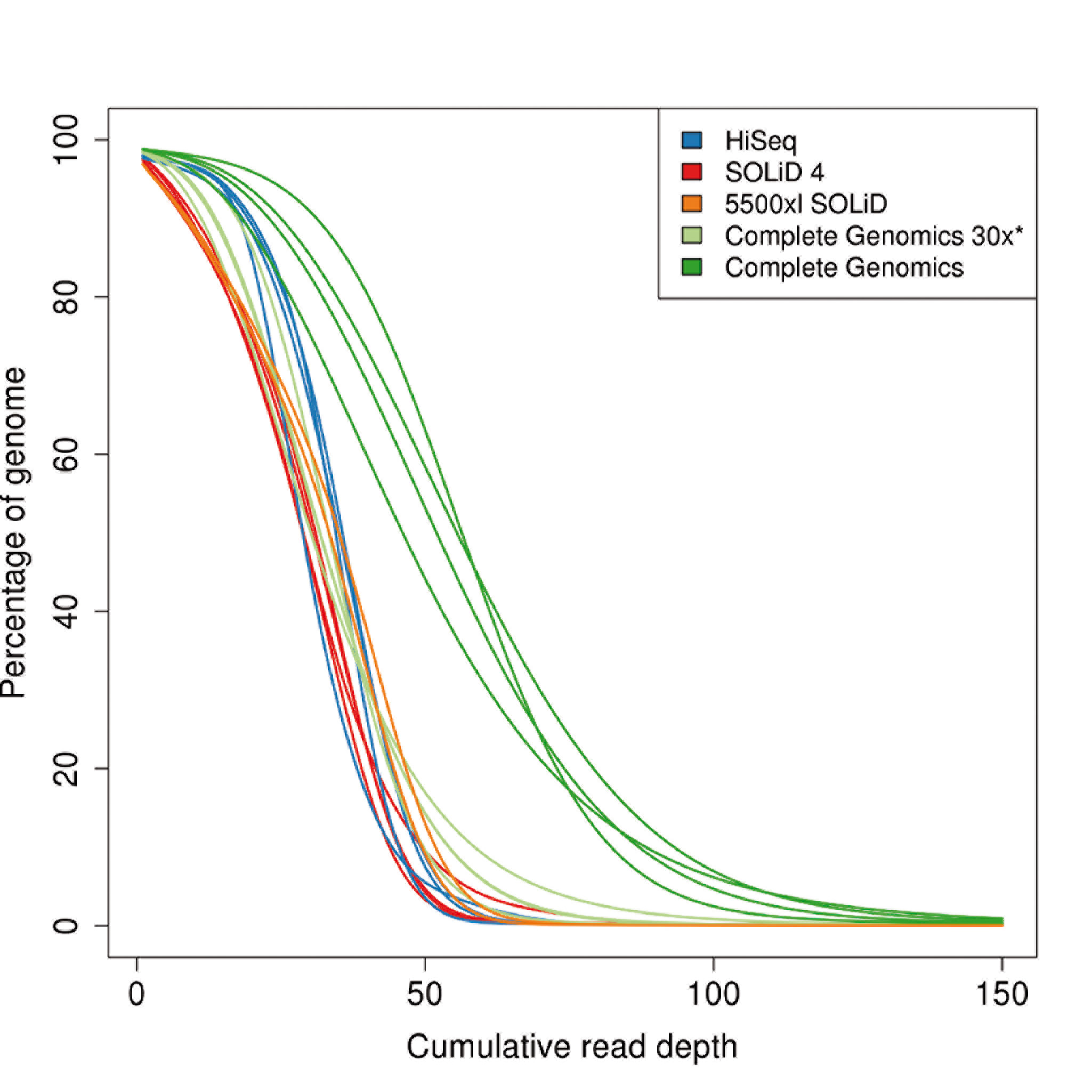

Supplement: Figure S6 — Cumulative base coverage distribution for the four platforms for all samples listed in Table 1 . Percentage of genome covered by read depth. Each curve corresponds to one sample. (TIFF) [file pone.0066621.s006.tiff]

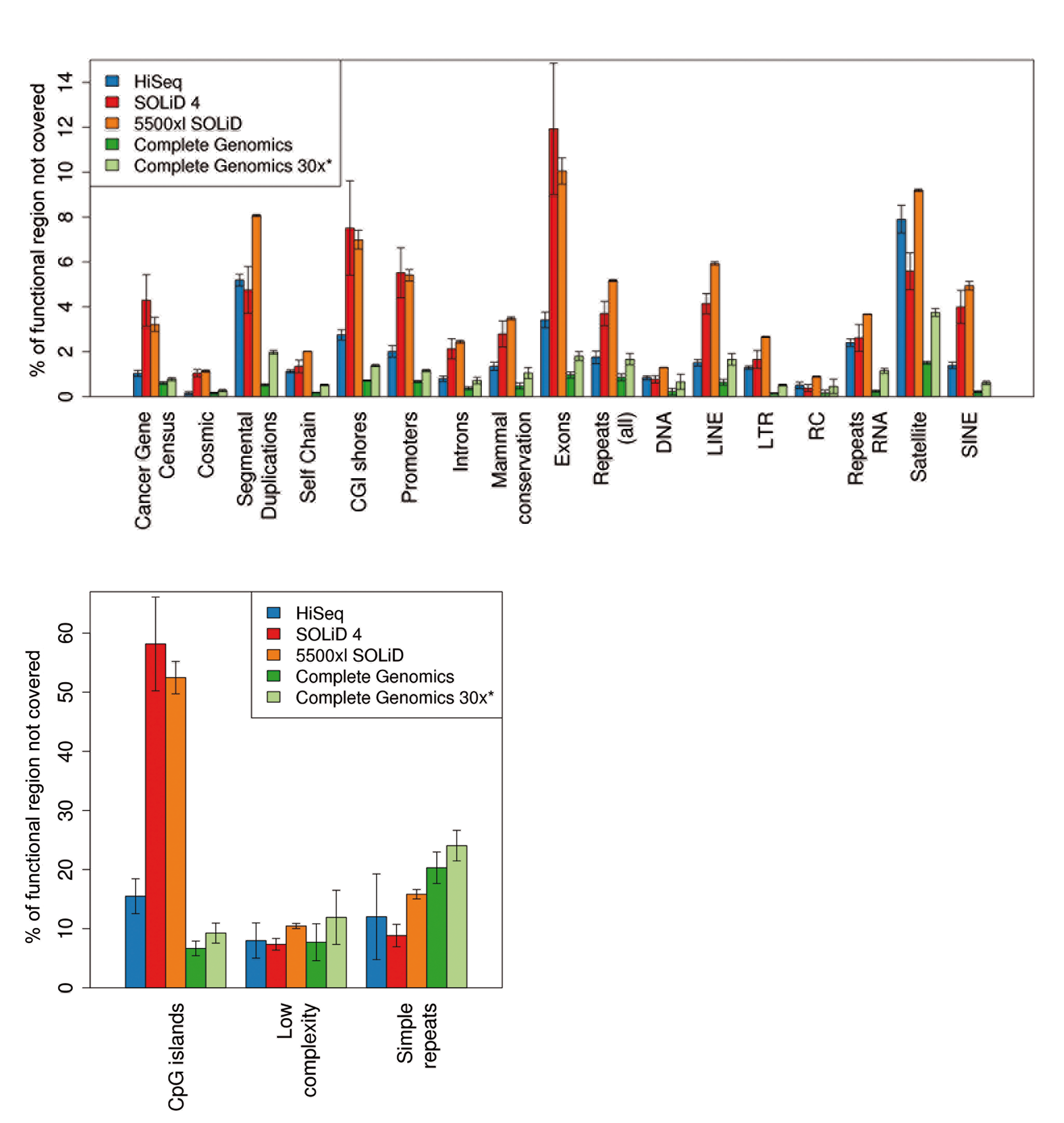

Supplement: Figure S7 — Percentage of bases without coverage across genomic elements, including Complete Genomics at full coverage. A base is considered not covered when it is covered by less than three reads. The error bars represent one standard deviation as obtained from analyzing the samples as listed in Table 1. DNA, LINE, Low complexity, LTR, RC, RNA, Satellite, Simple repeats and SINE are subcategories of Repeats (all). (TIF) [file pone.0066621.s007.tif]

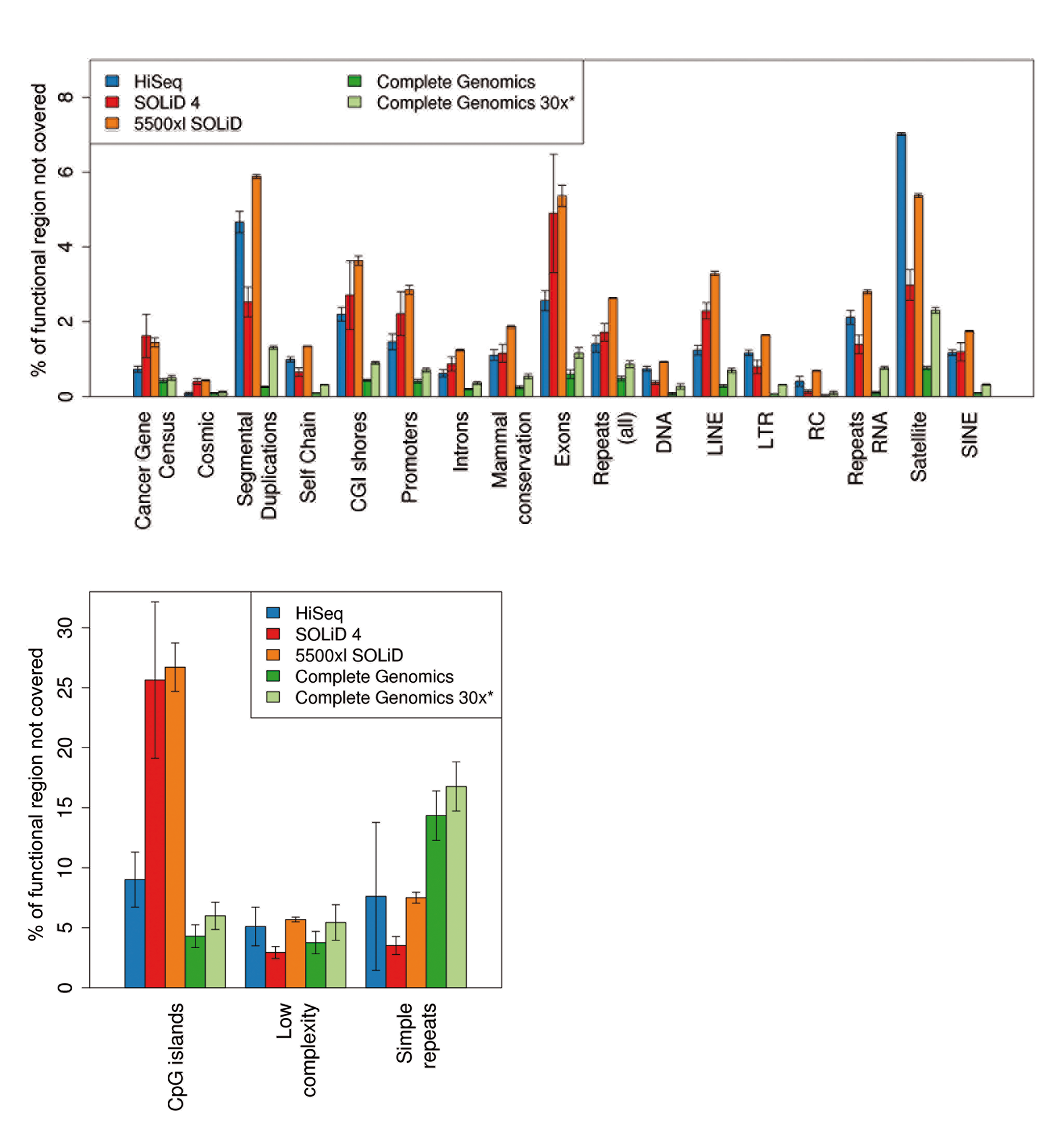

Supplement: Figure S8 — Percentage of bases without coverage across genomic elements. In this case, a base is considered not covered when it is covered by zero reads. The error bars represent one standard deviation as obtained from analyzing the samples as listed in Table 1. DNA, LINE, Low complexity, LTR, RC, RNA, Satellite, Simple repeats and SINE are subcategories of Repeats (all). (TIF) [file pone.0066621.s008.tif]

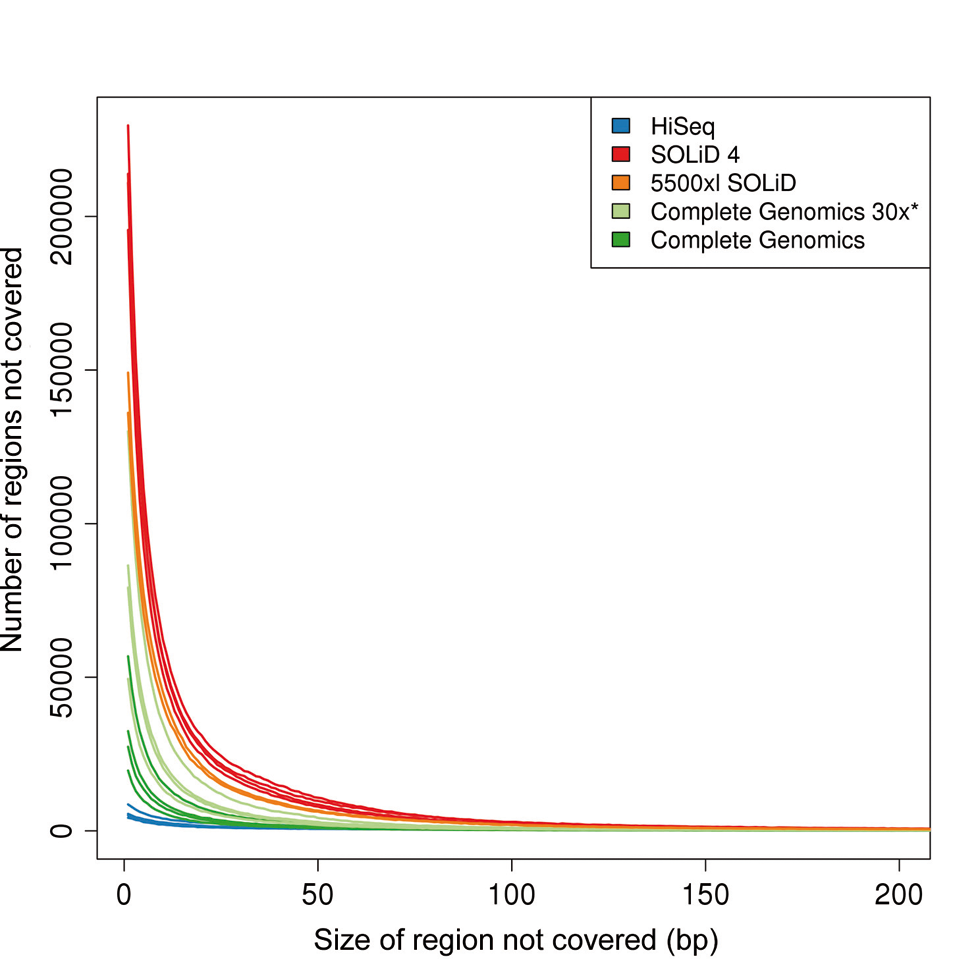

Supplement: Figure S9 — Size distribution of regions without coverage for all platforms and samples listed in Table 1 . Each curve corresponds to one sample. Based on the reference genome excluding N’s. A base is considered not covered when it is covered by less than three reads. The size of the largest region without coverage is approximately 110,000 bp in size for all four platforms, except for HiSeq (766,173 bp). This is due to the pseudoautosomal region on chrX/Y and is a consequence of mapping differences. (TIFF) [file pone.0066621.s009.tiff]

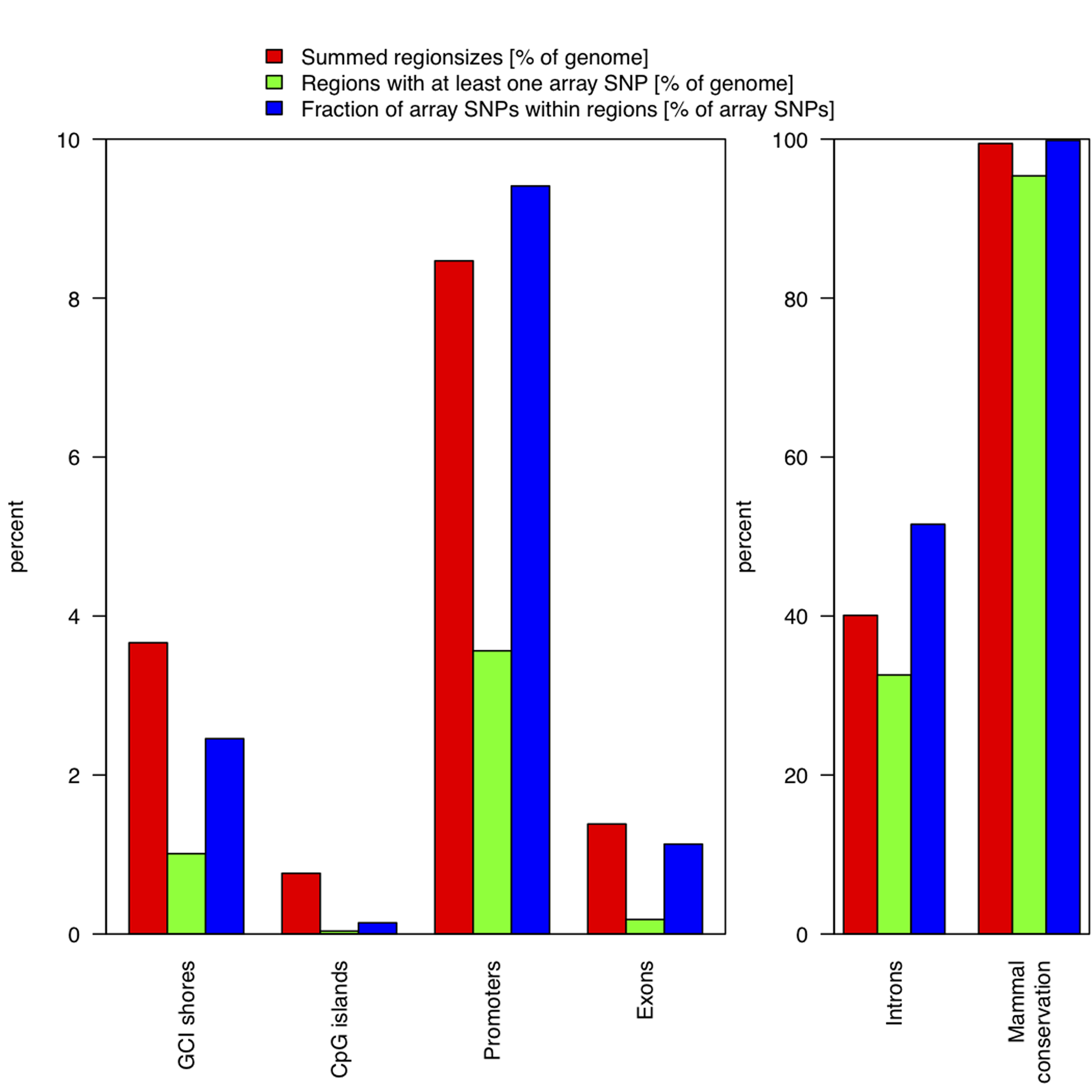

Supplement: Figure S10 — Distribution of Affymetrix SNP6 array SNPs in genomic elements analyzed. Percentage of genome covered by different types of genomic elements, in comparison to the distribution of SNP6 array SNPs on these genomic elements. (TIFF) [file pone.0066621.s010.tiff]

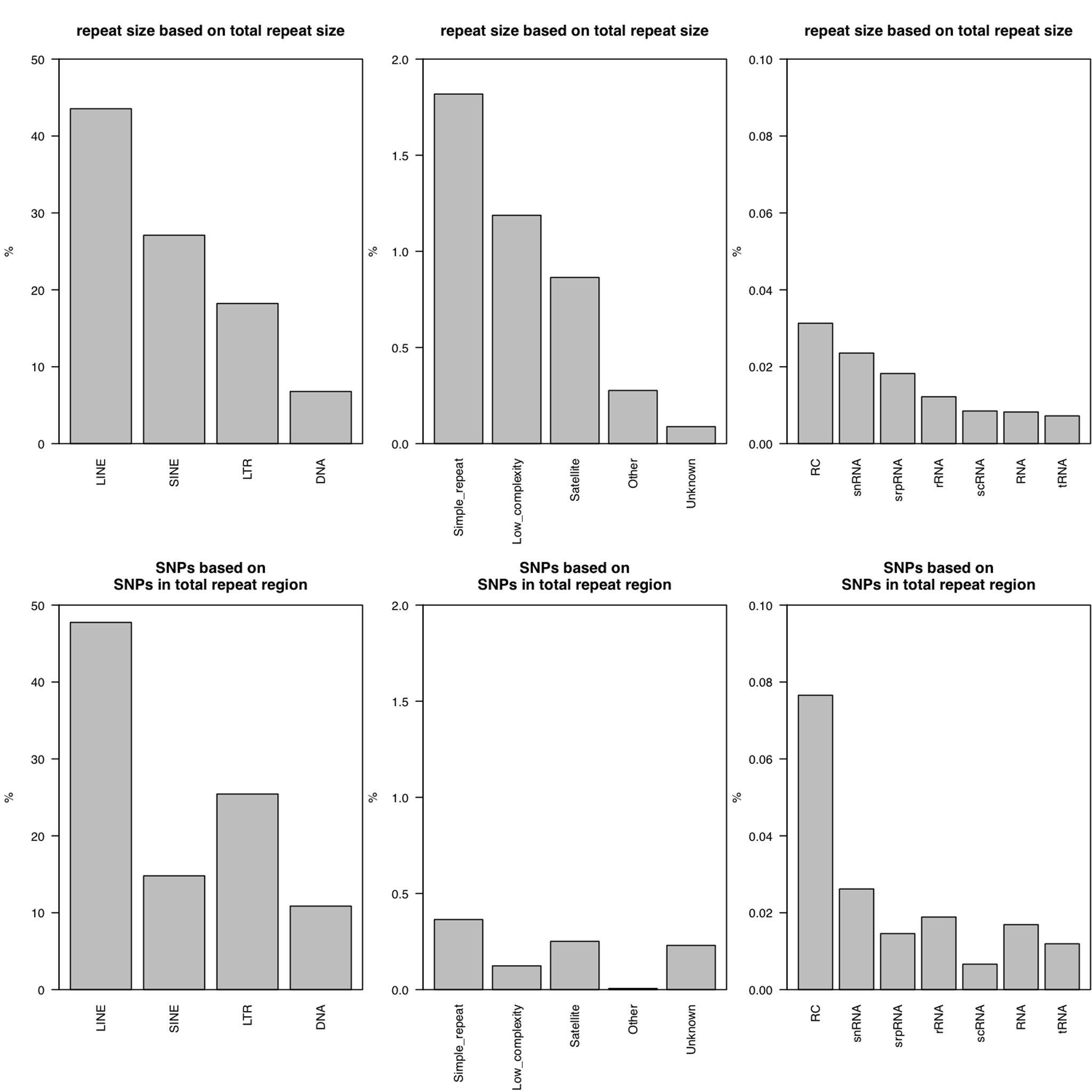

Supplement: Figure S11 — Distribution of Affymetrix SNP6 array SNPs in repeat types analyzed. The size of the different repeat regions was analyzed in comparison to the total repeat size. Overlapping repeat regions were reduced and not counted twice. All SNPs mapping to the repeat regions were identified and their distribution across the different repeat types compared to the total number of SNPs. (TIFF) [file pone.0066621.s011.tiff]

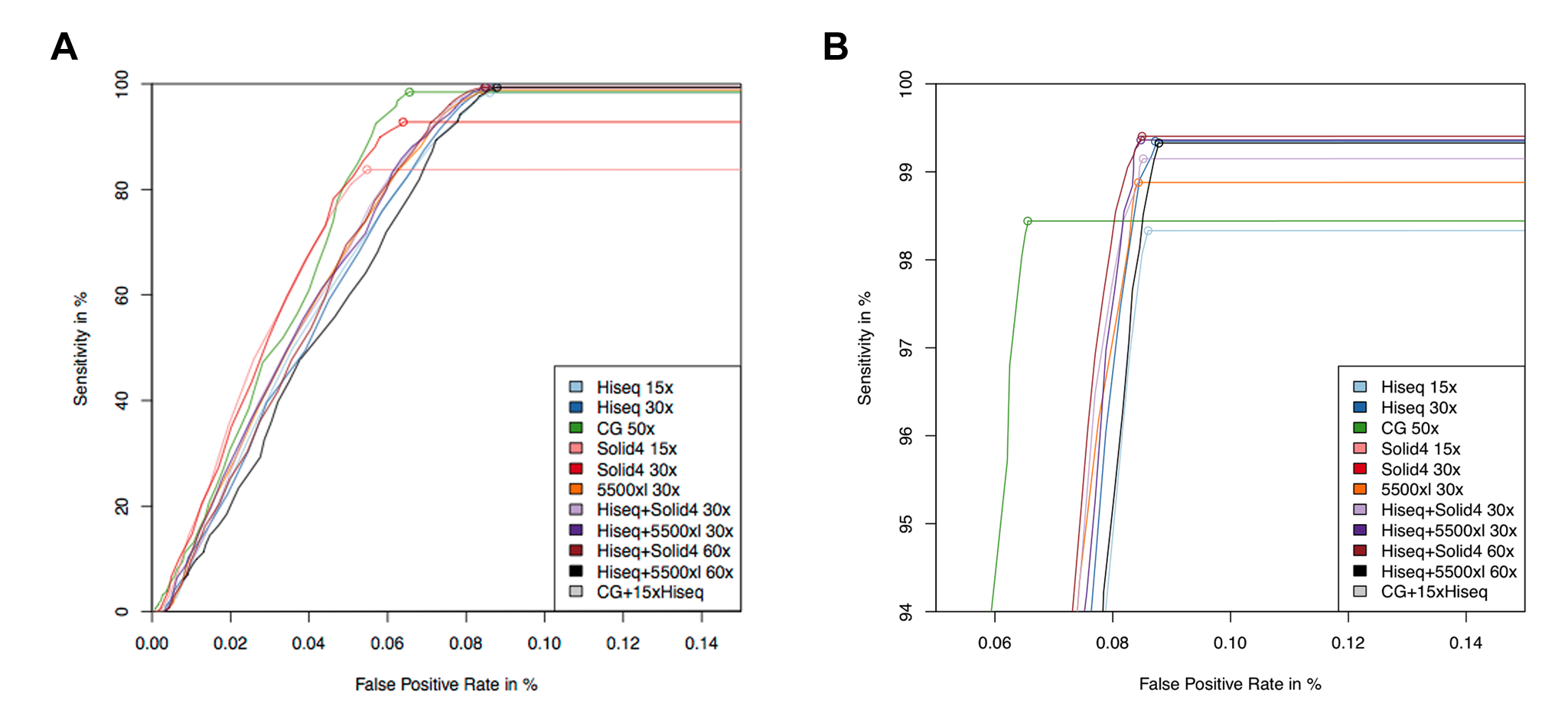

Supplement: Figure S12 — Receiver operating characteristic curves comparing sensitivity and specificity of all sequencing platforms for SNV calling. All curves are computed for exemplary patient sample BL24. When no additional coverage information is indicated, the curves are computed on full coverage data (for coverage information see Table 1). Additional numbers indicate either computationally downsampled data or combined data at specified additive coverage. (a) Specificity plotted from 0–0.17. All curves have reached their plateau at that point and will continue as straight lines. (b) Magnified view of curves to discriminate between subtle differences in specificity and sensitivity for all curves. Curves that do not appear in this magnified view reached their plateau below the cutoff of 94% sensitivity chosen for this window. (TIFF) [file pone.0066621.s012.tiff]

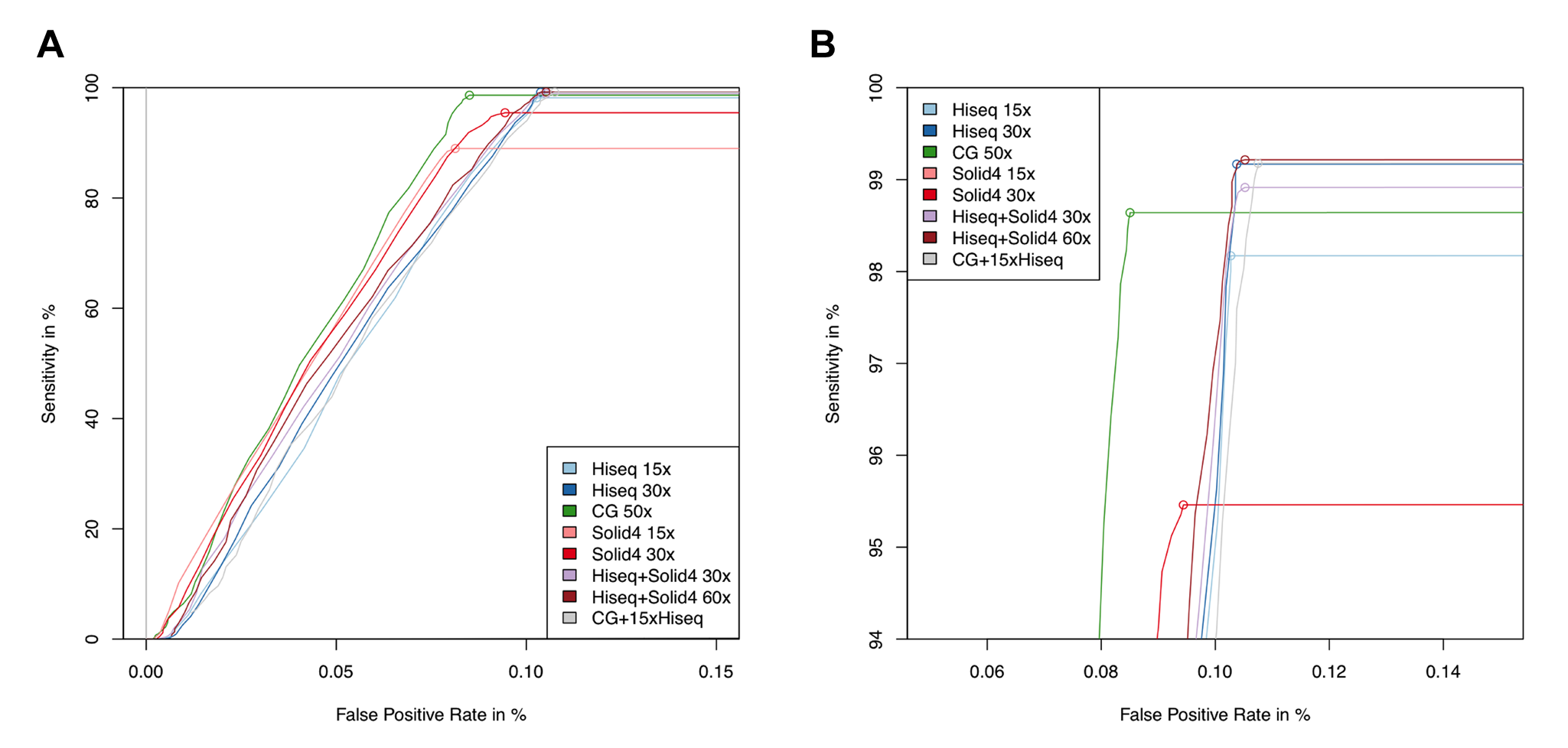

Supplement: Figure S13 — Receiver operating characteristic curves comparing sensitivity and specificity of all sequencing platforms for SNV calling. All curves are computed for exemplary patient sample BL14. When no additional coverage information is indicated, the curves are computed on full coverage data (for coverage information see Table 1). Additional numbers indicate either computationally downsampled data or combined data at specified additive coverage. (a) Specificity plotted from 0–0.17. All curves have reached their plateau at that point and will continue as straight lines. (b) Magnified view of curves to discriminate between subtle differences in specificity and sensitivity for all curves. Curves that do not appear in this magnified view reached their plateau below the cutoff of 94% sensitivity chosen for this window. (TIFF) [file pone.0066621.s013.tiff]

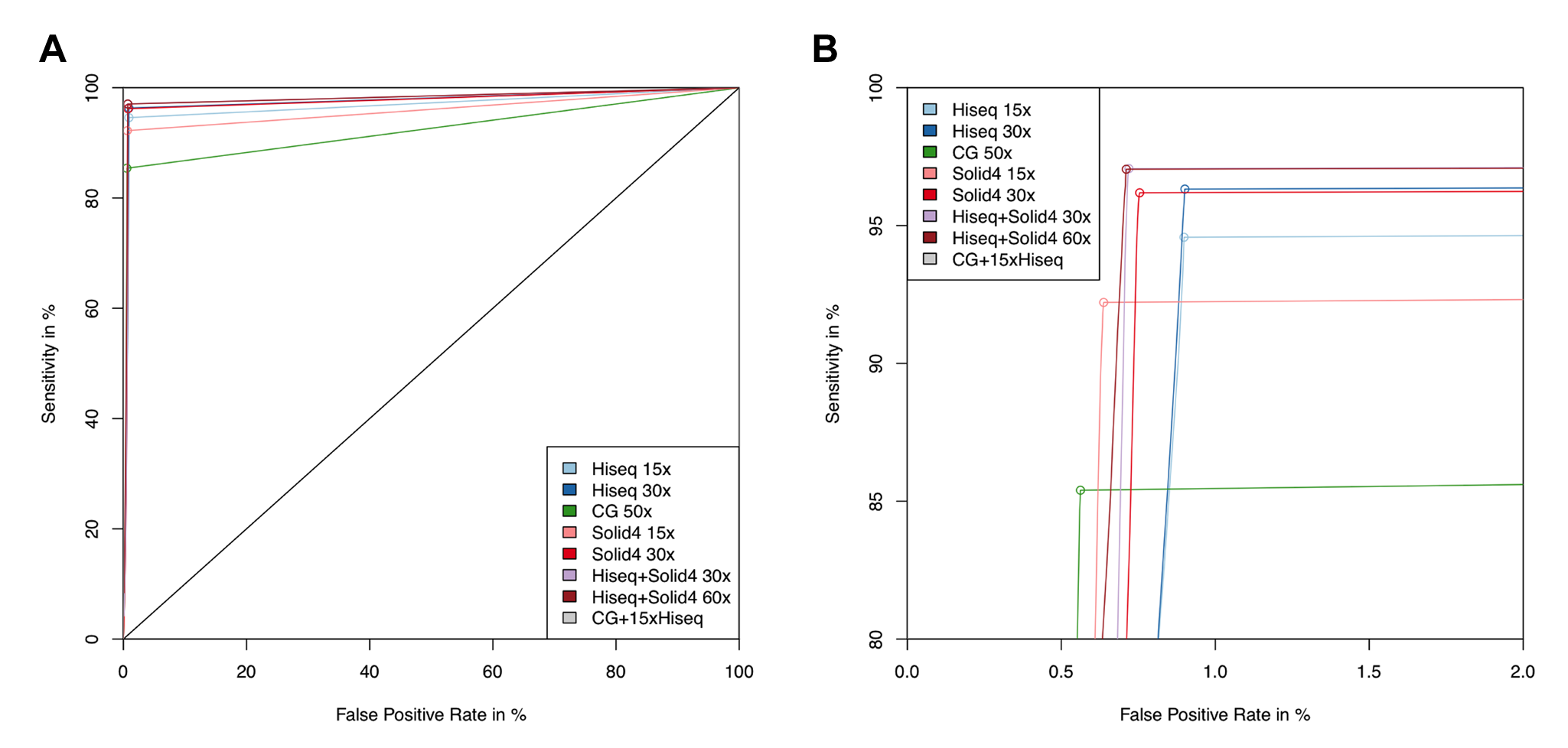

Supplement: Figure S14 — Receiver operating characteristic curves comparing sensitivity and specificity of all sequencing platforms for SNV calling. All curves are computed for exemplary patient sample MB14. When no additional coverage information is indicated, the curves are computed on full coverage data (for coverage information see Table 1). Additional numbers indicate either computationally downsampled data or combined data at specified additive coverage. (a) Specificity plotted from 0–0.17. All curves have reached their plateau at that point and will continue as straight lines. (b) Magnified view of curves to discriminate between subtle differences in specificity and sensitivity for all curves. Curves that do not appear in this magnified view reached their plateau below the cutoff of 94% sensitivity chosen for this window. (TIFF) [file pone.0066621.s014.tiff]
